# The Immunomodulatory Effect of *Trichophyton Rubrum* Exoantigens in the Treatment of Experimental Septic Arthritis

**DOI:** 10.2174/1874285801711010072

**Published:** 2017-05-30

**Authors:** Seyed. A Ghiasian, Amir H. Maghsood, Asadollah Abniki, Abbas Mirshafiey

**Affiliations:** 1 Medical Parasitology and Mycology Department, School of Medicine, Hamadan University of Medical Sciences, Hamadan, Iran; 2 Psoriasis Research Center, Department of Dermatology, Farshchian Hospital, Hamadan University of Medical Sciences, Hamadan, Iran; 3 Immunology Department, School of Public Health, Tehran University of Medical Sciences, Tehran, Iran

**Keywords:** Exoantigen, Septic arthritis, *Trichophyton rubrum*, Cartilage, Bone destruction, Synovial hypertrophy

## Abstract

**Background:**

Understanding the nature and function of fungal exoantigens might lead to novel approaches in the treatment and prophylaxis of some infectious diseases. Septic arthritis represents a serious problem for medicine due to the high incidence rate and severe complications.

**Objective:**

The present study aimed at assessing the immunomodulatory effects of *Trichophyton rubrum* culture filtrate as a novel compound in experimental septic arthritis.

**Method:**

The septic arthritis was haematogenously induced in Sprague-Dawley rats by a single intravenous injection of 10^9^ colony forming units of the human clinical isolate *Staphylococcus aureus* producing toxic shock syndrome toxin-1. *Trichophyton rubrum* culture filtrate at two different doses 20 and 40 mg/kg was administered intraperituneally two days after bacterial inoculation in the treatment groups and concurrently with the appearance of clinical signs in the patient groups. The administration of *Trichophyton rubrum* solution was continued every other day for 10 injections.

**Results:**

The clinical evaluation showed that *Trichophyton rubrum*-treated rats were significantly protected from disease development compared with untreated controls. This finding was correlated with results of radiological evaluation of the involved joints. Although, the inflammatory cell infiltration, cartilage/bone destruction and synovial hypertrophy had been decreased in the treatment groups in comparison with arthritic controls however, the histological changes were not significant in these two groups.

**Conclusion:**

It is possible that *Trichophyton rubrum* antigens may play a role in modulating the immune responses and would be efficient in septic arthritis treatment.

## 1 INTRODUCTION

Septic Arthritis (SA) or infectious arthritis is a serious condition characterized by direct invasion of joint space by various microorganisms such as bacteria, viruses and fungi [1]. Some studies have reported data on the incidence of SA in the Western Europe and USA, which respectively have been between 2 and 10 per 100,000 patient-years [2].

The dermatophytes include a group of medical and veterinary fungi which produce diseases basically limited to the skin, hair and nails. *Trichophyton rubrum* is an anthropophilic dermatophyte being the most common etiologic agent of dermatophytosis and widely distributed in the world. It uses human skin protein as a source of nutrient and regularly causes chronic infections of skin and nail and never cultured from soil [3].

Cell-mediated immunity is usually considered as the major immunological response to dermatophyte infections, characterized by the action of macrophages and some cytokines. Earlier, in crude culture filtrates of *T. rubrum* a number of proteinases such as acid proteinases, elastase, and keratinases have been identified to act as virulence factors [4]. There are several proteinases expressed by *T. rubrum* and its major proteolytic enzymes are M_r_ 93000 and 71000 [5]. There is some evidence that mannan produced by *T. rubrum* suppresses or reduces the inflammatory responses and causes chronic dermatophytosis [3]. Furthermore, it is proved that the 14,000 Mr extracellular serin proteinase purified from *T. rubrum* NP-9 culture filtrate has activities against gelatin, collagen type VI, and keratin [6].

According to Dahl and Grando reports, *T. rubrum* can make more mannan in comparison with other dermatophytes and its mannan is more potent immunosuppressor and possibly is able to suppress the reactions of cell-mediated immunity than mannans extracted from other fungi. Additionally, in the persistent form of infection, *T. rubrum* antigens induce immunological unresponsiveness by triggering specific suppressor T cells [7]. The various ways in which *T. rubrum* may contradict the immune system are lymphocyte inhibition by cell wall mannans, macrophage function modification by killing or modulating their activation, keratinocyte activation and the secretion of proteases. In spite of recent investigations, the mechanisms contributing to the immunomodulatory activities of *T. rubrum* cell wall mannans still poorly understood [8].

Generally, SA represents a serious problem for medicine due to the high incidence rate and severe complications. There is scarce data regarding the therapeutic effects of biological substances in inflammation-mediated diseases such as septic arthritis and in this regard, fungal constituents and their products were much less used. Therefore, search for new drugs effective against this human disease is of significant value. From this point of view and in order to develop the alternative strategies for treatment of this disease and search for new effective drugs, a preliminary study was conducted to evaluate the possible positive effects and immunotherapeutic ability of *T. rubrum* crude exoantigens on SA in the artificially infected rats.

## 2 MATERIALS AND METHODS

### 2.1 Rats

Forty outbred female Sprague-Dawley rats; 7-10 weeks old were obtained from Razi research Institute (Karaj, Iran) and housed in our animal facility (humidity, 55-56%; temperature, 20-22°C; 12/12 light/dark cycle; unlimited access to food and water) for at least 1-2 week before the experiment. Rats were kept in groups of five in each cage. Animals were divided randomly into three groups. P: patient group (n= 2 x 5); N: normal group (n=10); T1 (T1_A_ and T1_B_), and T2 (T2_A_ and T2_B_): treatment groups (each n = 2 x 5).

### 2.2 Bacterial Strain and Culture Conditions

*Staphylococcus aureus* (*S. aureus*) strain SHU-697, producing only toxic shock syndrome toxin-1 (TSST-1) was prepared from Shahed University culture collection. This strain was originally isolated from inflamed joint of a patient with rheumatoid arthritis. The production of enterotoxin was previously assessed using toxin detection kits (Denka Seiken Co. Ltd, Tokyo, Japan) for TSST-l and staphylococcal enterotoxins A-D (SEA-SED). In addition, TSST-l production was evaluated using an isoelectric focusing procedure [9, 10]. Bacteria were cultured on blood agar for 24 h, then reincubated on blood agar for another 24 h at 37°C. Bacteria were kept frozen at - 20°C in PBS until use. The bacterial solution was thawed and washed in physiological saline twice at the start of the experiment. Viable counts were used to check the number of live bacteria in each bacterial suspension [11]. To induce septic arthritis, a bacterial solution was prepared, using McFarland nephelometer no. 8 [12], and further diluted in physiological saline to the preferred concentration.

### 2.3 Culture Filtrate of *T. rubrum*

A clinical isolate of *T. rubrum* obtained from a patient with tinea pedis who referred to the Medical Mycology Department, School of Medicine, Hamadan University of Medical Sciences, Iran.

The isolate was cultured on Sabouraud’s dextrose agar (SDA) medium with chloramphenicol and cycloheximide (bioMérieux, Marcy-1, Etoil, France), which were made according to the manufacturer’s instructions. The plates were incubated at 28–30°C for 14–21 days and checked weekly. The dermatophyte grown on the plates were transferred to new SDA plates for pure culture and identified by standard methods including the gross morphology of the fungal colony and microscopic characteristics, especially according to their formation of macroconidia and microconidia in slide cultures. For differential diagnosis of *T. rubrum* and *T. mentagrophytes*, additional special methods including urea medium test, hair penetration, and production of pink color pigments on cornmeal agar medium plus 1% glucose were used. The isolate was then subcultured on to Sabouraud's agar culture medium (Oxoid, Basingstoke, UK), and then expanded into larger (250 ml and 500) volumes of Sabouraud liquid broth. For preparation of culture filtrate, *T. rubrum* was cultivated in a 500 ml flask containing Sabouraud liquid medium for 2 weeks at 28°C using a rotary shaker (120 rpm.). The cultures were autoclaved and reshaked. The mycelial mat and the filtrate were handled separately in a sterile condition and preserved by freezing at -20°C. The mycelial mat and the culture filtrate were separated under sterile condition by centrifuging at 6000 rpm for 30 min and filtering through a 0.2-pin sterile filter and preserved by freezing at -20°C. The filtrate was dialyzed in the cold against deionized water for 48 h. The culture filtrate used in this study was concentrated threefold, resulting in a carbohydrate concentration of 20 mg/ml, as determined by the phenol-sulfuric acid method [13] and then used immediately or stored at -20°C for no longer than 30 days.

### 2.4 Experimental Protocol and Treatment

The septic arthritis was induced as formerly explained [14]. In brief, 1 ml of an *S. aureus* suspension containing 1 × 10^9^ colony-forming units (CFU)/ml in physiological saline was intravenously injected in a lateral tail vein of each rat. In the control group, the same volume of physiological saline was given in the same method. Treatment groups (T1 and T2) received an intraperitoneal (IP) injection of *T. rubrum* culture filtrate, in doses of 20 and 40 mg/kg, based on carbohydrate concentration, after the appearance of disease (48-72 h). *Trichophyton rubrum* culture filtrate IP injections were continued at regular 48-h intervals for 10 injections.

#### 2.4.1 Clinical Evaluation of Arthritis

All rats were examined individually for the evaluation of weight, mortality and arthritis. Limbs were checked visually at regular intervals for up to 30 days. Arthritis was defined as visible erythema and/or swelling in the joints of either fore or hind limbs. Clinical scoring was performed based on the method of Abdelnour **et al.** [15]. Joint involvement was scored between 0 and 3 for each limb (0 point = normal appearance; 1 point = mild swelling and/or erythema; 2 point = moderate swelling and erythema; 3 point = marked swelling and erythema and occasionally ankylosis). To evaluate the intensity of arthritis, a clinical scoring (arthritic index) was used for each rat by dividing the total score (number of arthritic limbs) to the number of animals used in each experiment group. Furthermore, the general appearance and manners of the animals along with clinical evaluation of septic arthritis was carried by an independent blinded observer.

#### 2.4.2 Histopathological Evaluation

Histopathological assessment of the joints was carried out following sequential processing of fixation in formaldehyde, decalcification in paraffin embedding, as well as sectioning and staining with haematoxylin and eosin (H–E). Joint damage was evaluated based on inflammatory cell infiltration, synovial hypertrophy, cartilage and subchondral bone destruction and pannus formation (synovial tissue overlaying joint cartilage) [16]. Scoring was carried out in a blinded manner. Joint erosion was graded on a scale of 0-4 for each limb, according to the severity of damage.

#### 2.4.3 Radiological Evaluation

Radiological scoring was carried out on day 30 post bacterial inoculation by an independent investigator. A score was assigned to each joint on the basis of degree of soft tissue swelling, joint space narrowing, bone destruction and periosteal new bone formation. Scores were 0-3 per joint (0 point = normal; 3 point = maximum joint destruction) [17]. Radiology was accomplished in the School of Dentistry, Tehran University of Medical Sciences, Iran.

### 2.5 Statistical Analysis

Statistical analyses were performed using SPSS version 9.0 (SPSS Inc. Chicago, Illinois). Statistical comparisons were made using the Student’s t-test and Mann Whitney rank sum test. Data comparisons were considered significant if p<0.05.

## 3 RESULTS

**Fig. (1):**
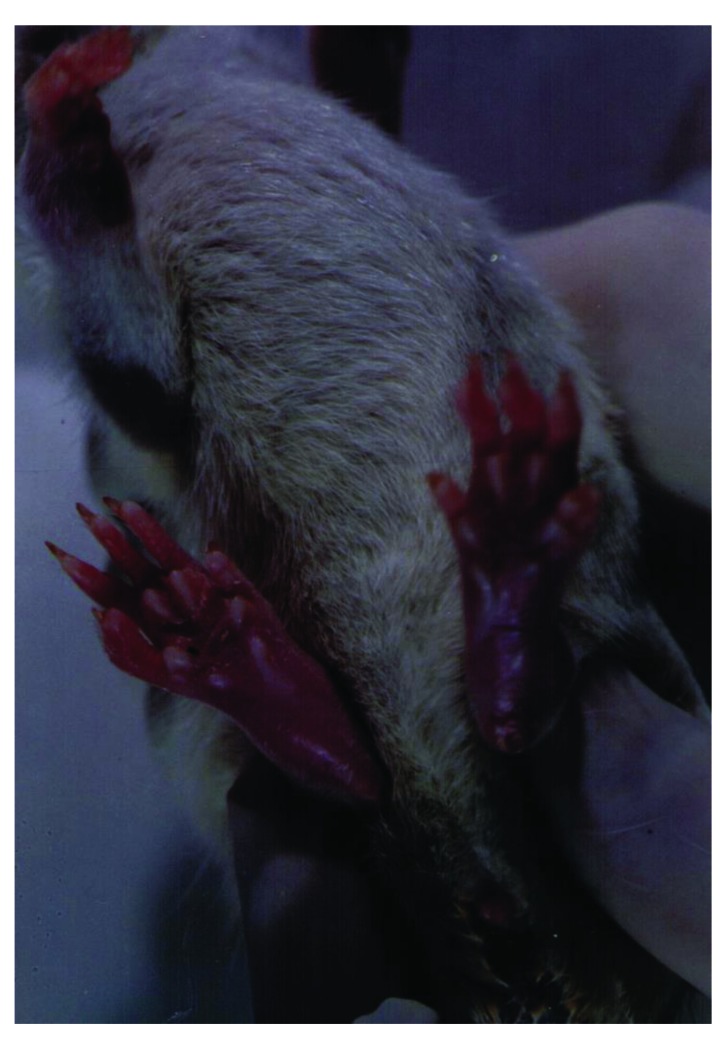
The severe swelling, inflammation and erythema of forepaw and hind legs joints in a rat model of septic arthritis.

**Fig. (2):**
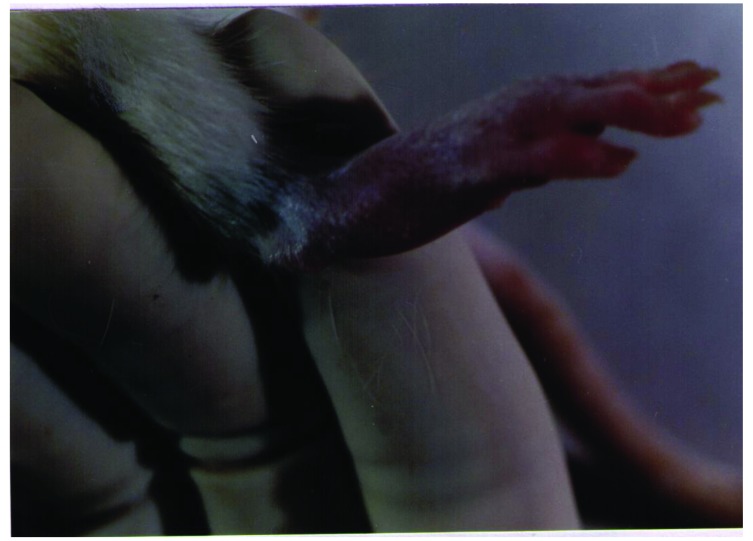
The severe swelling, inflammation and erythema of hind leg joints in a rat model of septic arthritis.

**Fig. (3):**
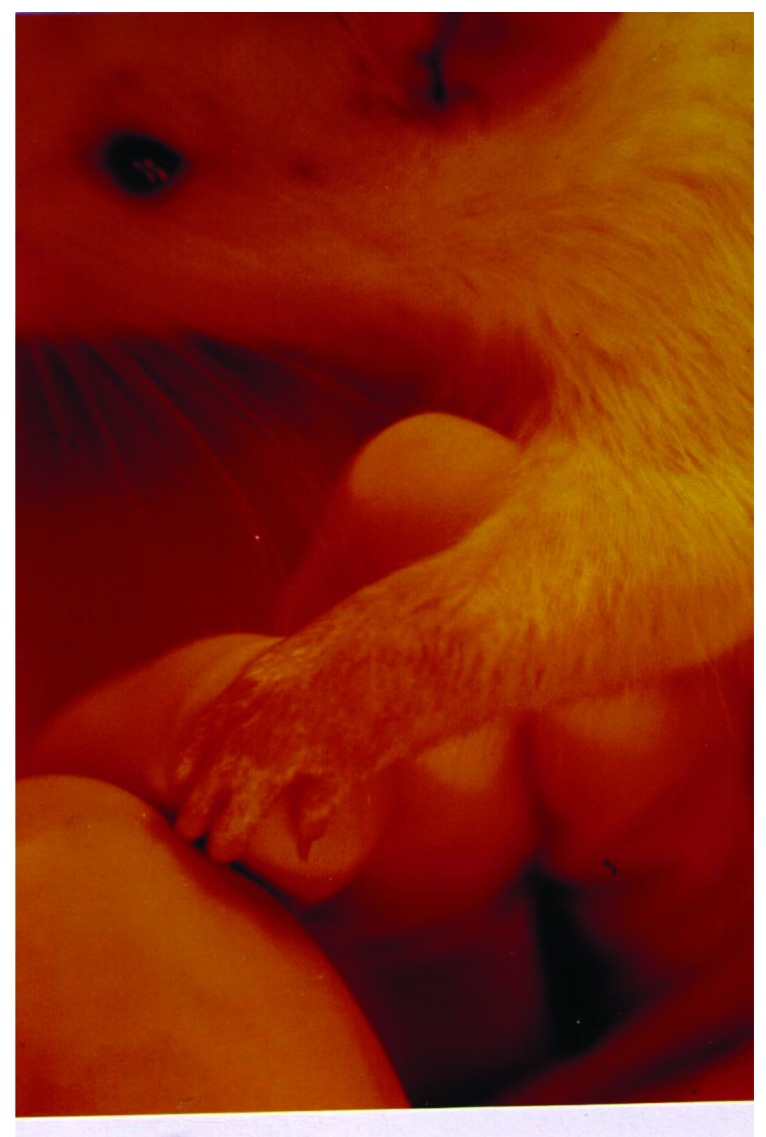
The severe swelling, inflammation and erythema of forepaw joints and palm in a rat model of septic arthritis.

**Fig. (4):**
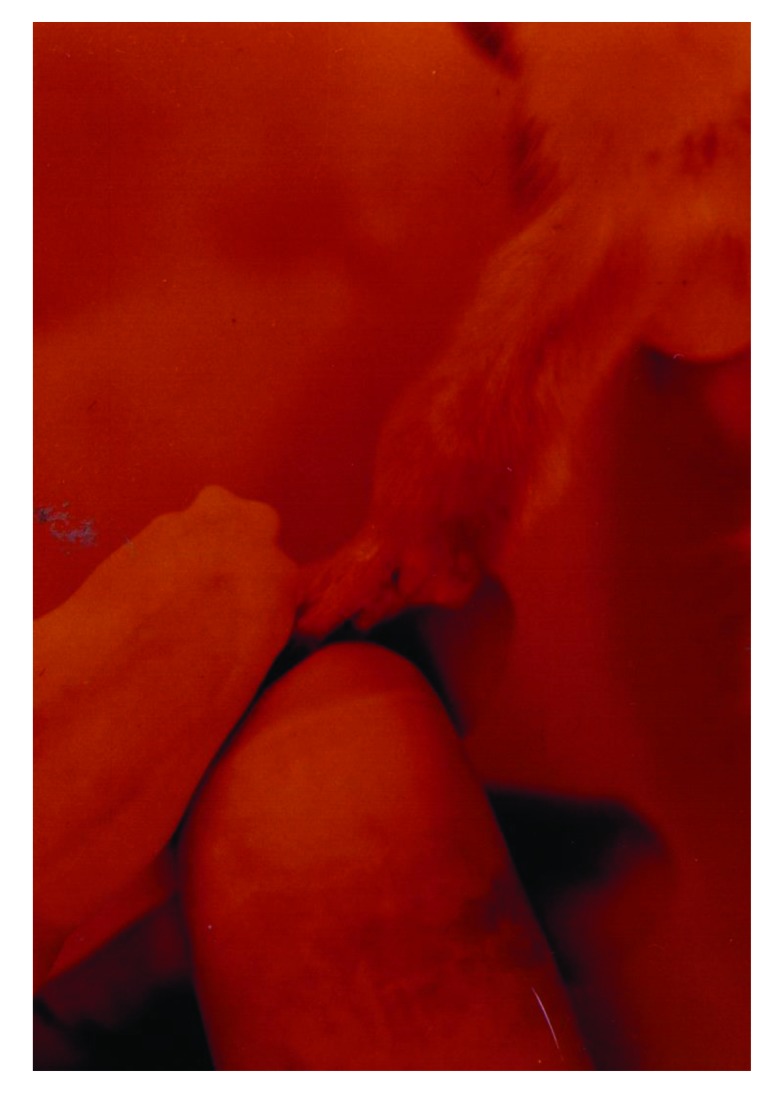
The severe swelling, inflammation and erythema of forepaw joints in a rat model of septic arthritis.

To set up the optimal infectious dose of *S. aureus* and the best way of administration, 40 Sprague-Dawley rats were studied in this experiment. The animals injected intravenously displayed the maximum proportion of arthritis, especially those given 10^9^ bacteria. Thus, all of the rats injected with 10^9^ bacterial cells displayed clinical signs of septic arthritis, which included joint swelling concomitant with mild to severe joint erythema, on day 20 and continued through to the last day 30. The sign of swollen joints was prominent especially after 10 days of the experiment and hindpaws and forepaws were generally affected (Figs. **1**-**4**).

**Fig. (5):**
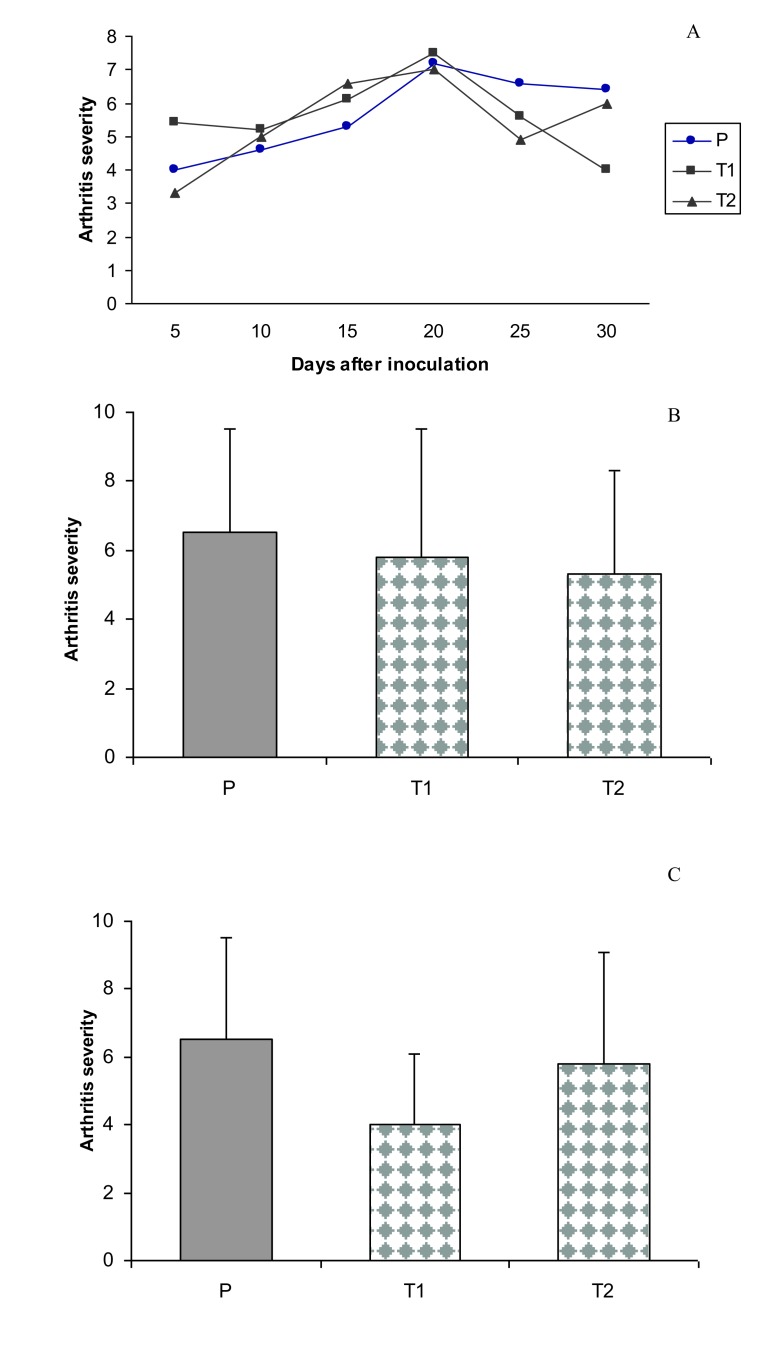
Anti-arthritic effect of *T. rubrum* in different groups. P: patient rats and Tl, T2: Treated patient rats. Septic arthritis was induced on day 0 by iv. injection of l0^9^*S. aureus* per rat. Tl, T2 rats received 20 and 40 mg/kg *T. rubrum* by IP injection on day 3. The total number and intervals of injections were 10 and 48h, respectively. Bars show the mean ± SD. **(A)** Time course of mean values of clinical scores of septic arthritis in groups P, Tl and T2. **(B)** Effect of *T. rubrum* on arthritis severity in different groups on day 25 (4 days after the end of *T. rubrum* IP injections), P versus T1 were significant (*P* <0.05). **(C)** Anti-arthritis effect of *T. rubrum* in different groups on day 30 (9 days after the end of *T. rubrum* IP injections), P versus T2 were significant (*P <*0.05).

The results of the current experiment for the first time clearly demonstrated that treatment with *T. rubrum* was able to significantly reduce the clinical signs and severity of arthritis in treated rats (T1) compared with non-treated animals (*p*<0.05) (Fig. **5**).

**Fig. (6):**
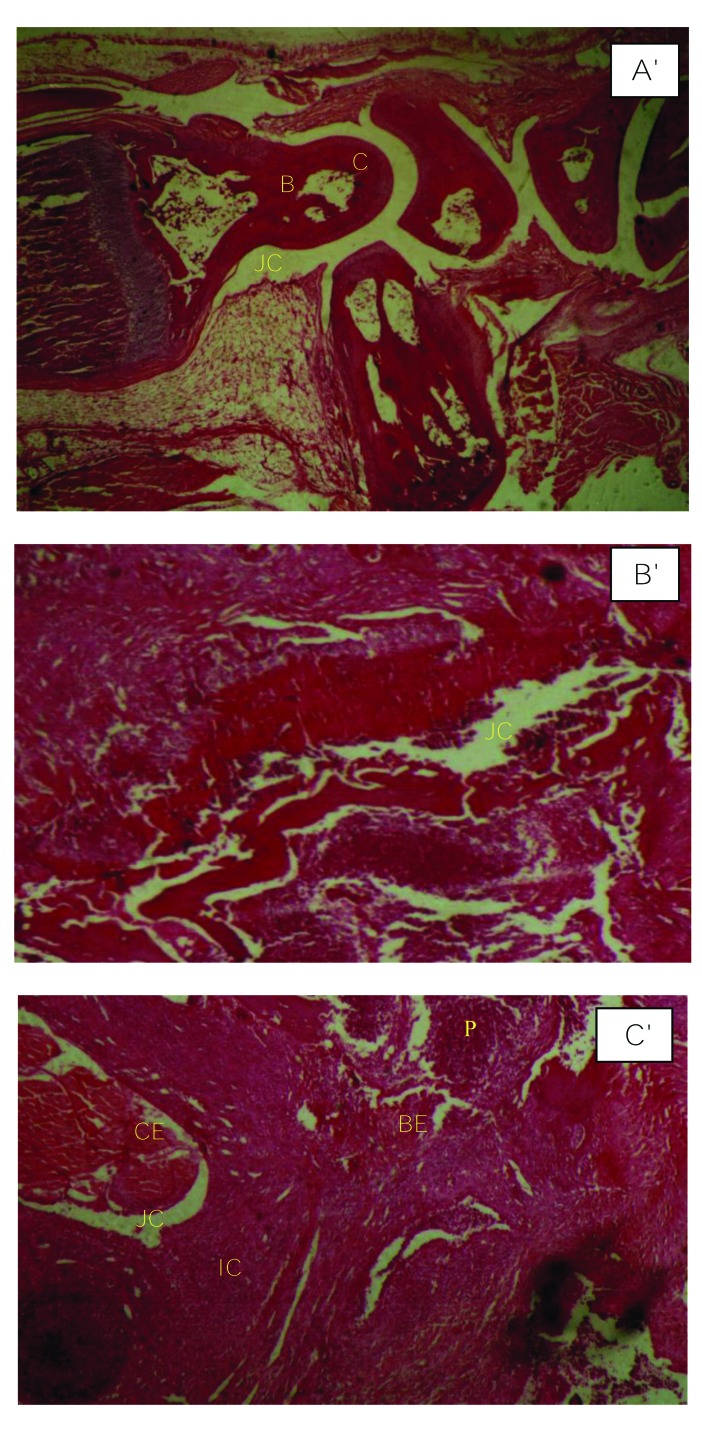
Hematoxylin-eosin stain preparation of organ sections. Histopathological analyses in a hind paw of normal rat **(A')**; rat model of septic arthritis **(B')**; and leg joint in a treatment group (T2) rat **(C'),** C: cartilage; B: bone; BE: bone erosion; CE: cartilage erosion; JC: joint cavity; IC: inflammatory cells and P: pannus formation. Histopathological micrographics are shown with 10x magnification.

Histopathological features of swollen joints in patient rats demonstrated inflammatory cell infiltration, synovial tissue proliferation, hypertrophy and destruction of both cartilage and subchondral bone, together with pannus formation. *T. rubrum* culture filtrate therapy with doses of 20 mg/kg and 40 mg/kg in the therapeutic groups showed that this anti-inflammatory substance could not significantly reduce the histological changes in treated rats compared with non-treated animals (Fig. **6**).

**Fig. (8):**
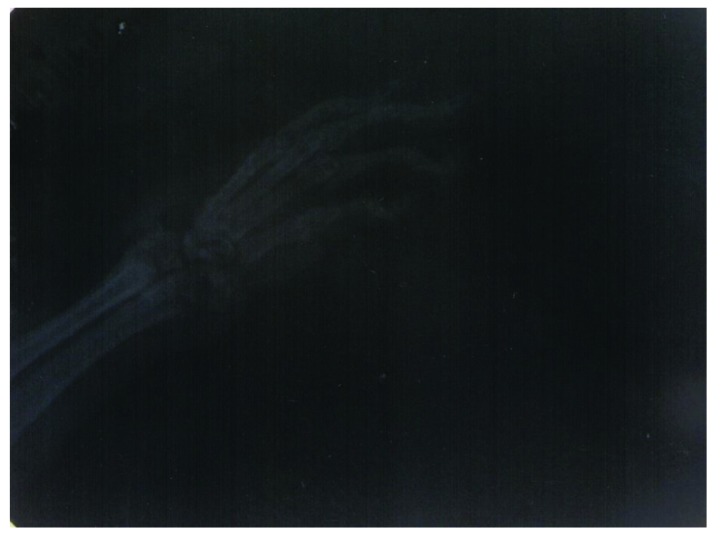
The inflammation of the forepaw joints in a rat model of septic arthritis (radiographical score 3).

**Fig. (9):**
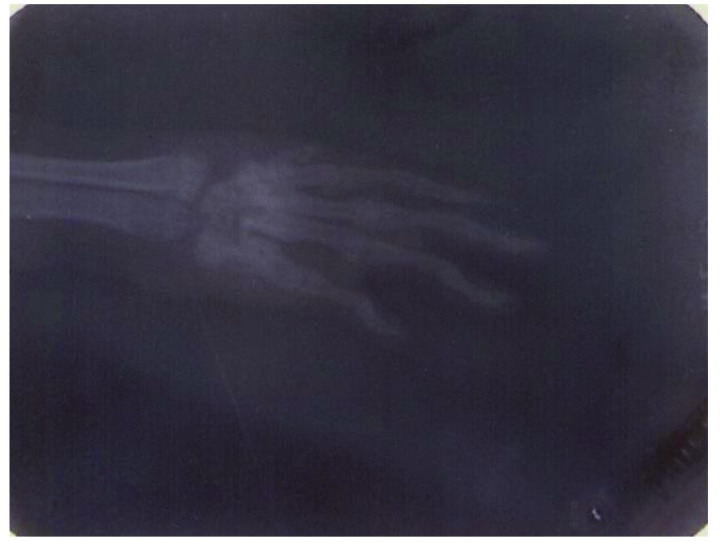
The inflammation of the forepaw joints in a rat model of septic arthritis (radiographical score 4).

Radiographical features of affected joints in patient rats demonstrated joint space narrowing, soft tissue swelling, reduced lucency due to demineralization and areas of recalcification indicating new bone formation. Blinded radiographical scores (0-4) on day 28 were significantly lower in both groups of *T. r*-treated rats (T1_A_, T1_B_, and T2_A_, T2_B_) than patient groups Fig. (**7**). The radiographical appearance of swollen joints was comparable with the results of arthritic index and histological changes of the joints (Figs. **8**, **9**).

## 4 DISCUSSION

To our knowledge, the present preliminary study is the first report contributed to the antiarthritic and therapeutic effects of IP administration of different doses of *T. rubrum* exoantigens in a rat model of SA in order to develop the alternative strategies for treatment of this disease.

Septic arthritis is the most common type of infective arthritis and since there is still lack of efficient cure for this disease, a serious necessity for a novel therapeutic agent able to inhibit the disease progression and joint destruction will be required. Understanding the nature and function of fungal exoantigens is an exciting challenge that might lead to novel approaches in the treatment and prophylaxis of some infectious diseases. In this respect, the dermatophytic fungi, because of their antigenic nature, are seemed to be appropriate for this purpose. The dermatophytes may inhibit the immune system through different methods including lymphocyte inhibition *via* cell-wall mannans as well as alteration and modification of immune cell function. Although the development of humoral immunity with higher antibody titers in chronic dermatophytic infections has been established, it has not generally been efficient in overcoming the infection [18-20]. Furthermore, previous achieved results showed that an interaction between *T. rubrum* conidia and resident macrophages results in the production of TNF-a and IL-10. It has also been shown that phagocytosis of *T. rubrum* conidia is prohibited by adding of fungal exoantigen mannan [21].

It is currently accepted that cell-mediated immunity is responsible, in general, for the control of dermatophytoses and resistance to these infections results from proficient priming of Th1-mediated cellular immunity [22]. Furthermore, in contrast to acute and highly inflammatory dermatophyte infections, a negative delayed-type hypersensitivity response to an intradermal injection of trichophytin has been demonstrated in individuals with chronic dermatophytosis [23].

A few numbers of dermatophytes, like *T. rubrum*, are extremely adapted to human and able to evade or calm the immune response by killing macrophages or modulating their activation program, consequently causing chronic, recurrent and relatively uninflamed dermatophytosis. One of the main reasons in terms of this chronicity is the immunosuppressive effects of galactomannan, one of the major cell-wall components.

*Trichophyton rubrum* cell-wall mannans are able to inhibit lymphoproliferative response of mononuclear leukocytes to several dermatophytic and non dermatophytic antigens as well as mitogenic stimulation *in vitro* [24]. Taking into account the mentioned considerations, *T. rubrum* cell-wall mannans seem to be implicated in an immunosuppression phenomenon.

Despite numerous investigations on the pathophysiological mechanisms of mycoses, especially dermatophytes, the immunomodulatory or immunotherapeutic properties of fungal antigens remains poorly understood.

The principal component of fungal cell walls, (1→3)-β-d-glucans, have been shown to have motivating effects on the defense mechanisms of the living organisms by increasing the host resistance to fungal, bacterial, viral, and parasitic infections as well as to neoplastic and other pathogenic situations [25]. An earlier study reported that mannan from *Candida albicans* prohibits both inflammation and destructive arthritic changes, thus the possible immunoregulatory and therapeutic effects of this simple carbohydrate were demonstrated [26]. According to Prokopová *et al.*, the mannose present in yeast mannans are capable of mediating *in vitro* suppression of antigen driven T-lymphocyte proliferation, hexose monophosphate shunt activation as well as prostaglandin and IL-1 synthesis [26]. In this regard, Sathyamoorthy, *et al.*, suggested a theory that specific mannose saccharide structures could contribute, *in vivo*, to the physiologic regulation of immune response [27]. This interesting point was considered by Mirshafiey *et al.*, who suggested that the culture filtrate of *Cryptococcus neoformans var. gattii* (CneF) may have therapeutic potential in the treatment of septic arthritis as well as can reduce proteinuria, suppress the development of glomerular lesions, and exert lipid-lowering property in a rat model of immune complex glomerulonephritis [14, 28]. Taking all these facts into account, in the current study we investigated the immunotherapeutic ability of *T. rubrum* exoantigens to reduce the clinical signs and severity of septic arthritis in the artificially infected animals. Of interest, a similarity to our results has been found in the study conducted by Mirshafiey *et al.* [28], where glucuronoxylomannan and galactoxylomannan, the major capsular polysaccharides of *C. neoformans*, illustrated both anti-inflammatory and immunosuppressive properties in experimental *S. aureus* arthritis. They concluded that “therapeutic effects of CneF could be due to biochemical characteristics of major (mannan and glucuronic acid) and minor (galactose and xylose) determinants in the molecular structure of CneF components (GXM and GalXM)”. They also believed that the GXM molecule because of some characteristics such as mannose backbone structure, high negative charge and polyanionic properties can accelerate the therapeutic effects of this molecule. Such findings match the theory proposed by Kéry [29], whereby yeast cell wall mannans could exert various immunoregulatory properties.

In the present study, the significant statistical difference in radiological examination of the involved joints in the patient and treatment groups was in agreement with that earlier described by Mirshafiey *et al.* [28]. However, in histological assessment, no significant difference was found between these two groups. These results were in contrast to those of Mirshafiey *et al.* [28], who reported that culture filtrate of *Cryptococcus neoformans* var.* gattii* could significantly reduce the histological changes in treated animals in comparison with non treated ones.

## CONCLUSION

In conclusion, these promising results about the anti-inflammatory and immunotherapeutic effects of *Trichophyton rubrum* exoantigens as well as formerly obtained evidence on the anti-inflammatory materials of some other fungi led us to realize that fungal antigens would elicit their protective effects in the experimental model of septic arthritis. Overall, the efforts have been made for new drugs against SA are encouraging, however there are still some gaps to be filled.

## Figures and Tables

**Fig. (7):**
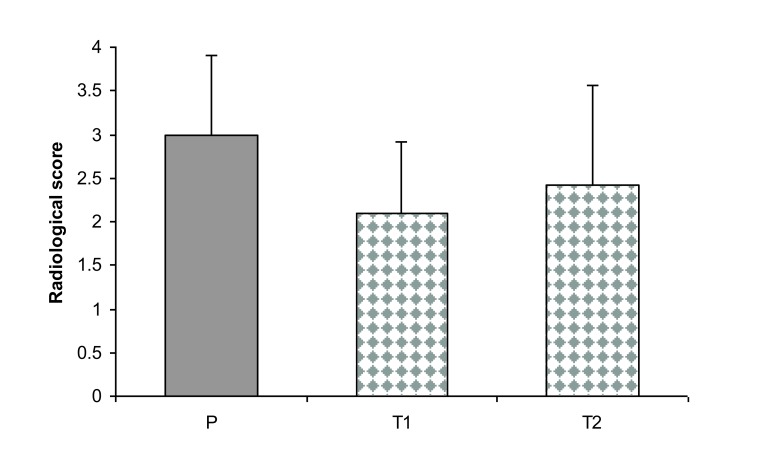
Semi-quantitative scoring of radiological examination of the involved joints in groups P (patient) and T1 and T2 (treatment). P versus T1 and T2 were significant. Bars show the mean ± SD. *P* <0.05 was considered statistically significant.
